# Knowledge of pelvic floor disorders in women seeking primary care: a cross-sectional study

**DOI:** 10.1186/s12875-019-0958-z

**Published:** 2019-05-23

**Authors:** Chi Chiung Grace Chen, Jacob T. Cox, Chloe Yuan, Lauren Thomaier, Sonia Dutta

**Affiliations:** 1 Department of Gynecology and Obstetrics, Johns Hopkins Hospital, Baltimore, MD 21224 USA; 2 Johns Hopkins University School of Medicine, Baltimore, MD 21205 USA; 3 John A. Burns School of Medicine, Honolulu, HI 96813 USA

**Keywords:** Pelvic floor disorder, Urinary incontinence, Vaginal prolapse, Awareness, Knowledge

## Abstract

**Background:**

Pelvic floor disorders including urinary incontinence (UI) and pelvic organ prolapse (POP) are common conditions; however, most women with these symptoms do not seek care. Failure to seek care may be related to misconceptions about these conditions. The aim of this study was to assess the baseline knowledge of UI and POP among adult women presenting to primary care clinics, as well as factors associated with knowledge levels.

**Methods:**

A survey with questions from previously validated UI and POP knowledge questionnaires (PIKQ-UI and PIKQ-POP, respectively) was self-administered to a cross-sectional group of adult female patients presenting to three primary care clinics: geriatric, community-based, and hospital-based. Participants’ demographics and medical histories were compared using ANOVA or Kruskal-Wallis for continuous variables and Chi-square test or Fisher’s exact test for categorical variables. In order to compare various covariates with knowledge non-proficiency on PIKQ-UI and PIKQ-POP scales, unadjusted and adjusted ORs with 95% CIs were calculated using bivariate analysis and multivariate logistic regression, respectively.

**Results:**

Of 346 participants, knowledge non-proficiency was similar and consistent across clinic sites and reached 72.0% for UI and 53.6% for POP. On multivariate analysis, lower educational attainment, being unaware of UI or POP as medical conditions, and having no history of care-seeking for these conditions were significantly associated with knowledge non-proficiency on UI, POP, or both.

**Conclusions:**

Knowledge non-proficiency for UI and POP is common among women presenting for primary care. For UI, healthcare providers should assess patients’ actual understanding of the disease, especially among those with lower educational attainment, to eliminate any possible misconceptions. For POP, the focus should be on increasing awareness of this disease, as many women may have not previously heard of this condition. Simple strategies may increase knowledge in these areas and change care-seeking behaviors.

**Study registration:**

None.

## Background

Pelvic floor disorders (PFDs) consist of urinary incontinence (UI), fecal incontinence (FI), and pelvic organ prolapse (POP). The prevalence of PFDs is estimated to range between 12 and 42%, and while symptoms can present as early as age 20, their prevalence increases with age and they are most often seen in late adulthood [1, 2]. Thus, as life expectancy continues to increase in developed nations and the elderly female population grows, PFDs are expected to become more prevalent [3, 4]. This upsurge has already led to increases in PFD-related healthcare costs. Two decades ago, national PFD-related costs were roughly $12 billion per annum [5]. By 2007, this sum had risen to $66 billion per annum and continues to increase dramatically [6, 7]. These conditions can diminish one’s quality of life in various ways, including social isolation and psychological distress. Despite these social and fiscal consequences, fewer than half of women with significant PFD symptoms seek care [8–10].

Research has indicated that this failure to seek care is related to misconceptions about the conditions themselves, with one study demonstrating that 81% of women do not perceive UI as abnormal and believe that PFDs are a “natural part of childbirth and aging” [9, 11–14]. Additional barriers to presentation include personal embarrassment, being unaware that PFDs are medical conditions, and being unaware that treatment options exist [10, 11, 15–17]. However, studies addressing patients’ knowledge of PFDs have primarily focused on women presenting to urogynecologic specialists for care [18–21]. While understanding PFD knowledge in women presenting to specialty clinics is important, gauging the knowledge gap among more general female populations is arguably more pertinent. This gap is particularly critical to assess given that early treatment of PFDs enables use of conservative, non-surgical treatment options, such as Kegel exercises or pessary placement, which have demonstrated efficacy and may delay disease progression [22, 23].

However, little is known about the PFD knowledge gap among female populations not already presenting for specialty care. As such, the objective of this study was to determine baseline levels of PFD knowledge among adult women presenting to primary care clinics. Our secondary objective was to assess associations between gaps in PFD knowledge and various clinical or demographics factors. Through these findings, we hope to guide efforts by primary care providers (PCPs) and other generalists to better inform their patients about these conditions, which may lead to changes in care seeking behaviors.

## Methods

A cross-sectional, self-administered survey was conducted to assess PFD knowledge among women presenting to three primary care clinics: a hospital-based general internal medicine clinic, a community-based general internal medicine clinic, and a geriatric medicine clinic. The study was limited to English-speaking women over the age of 18. No unique patient identifiers were collected, and Institutional Review Board (IRB) approval was obtained from Johns Hopkins University School of Medicine (IRB NA_00089290) prior to study initiation. Women were recruited using two techniques: (i) team members approached and invited them to complete a written questionnaire, or (ii) they elected to pick up a questionnaire at advertised locations in the various clinics. Regardless of how participants were approached, all participants ultimately completed the questionnaire on their own. The questionnaire addressed demographics, medical history, sources of healthcare information, awareness of PFDs as a medical condition, and questions from a previously validated knowledge assessment tool called the Prolapse and Incontinence Knowledge Questionnaire (PIKQ) [18]. Upon completion, participants returned surveys to medical assistants or designated areas.

As previously described by Shah et al., PIKQ is a 24-item questionnaire with 12 questions related to UI epidemiology, pathogenesis, diagnosis, and treatment (PIKQ-UI) and 12 questions related to these same parameters in POP (PIKQ-POP) [18]. Only correct responses were counted, with each correct response receiving one point. Knowledge proficiency, indicating higher than usual knowledge, was established as ≥ 80 and ≥ 50% for PIKQ-UI and PIKQ-POP, respectively, based on frequency data from the original study authors [18]. This allowed for dichotomization between knowledge proficiency and knowledge non-proficiency, which was used in all subsequent analyses. We chose to use these same cut-off values since they have been used in multiple prior publications and would thus allow for inter-study comparability.

Data were entered manually into Microsoft Excel 2007 (Microsoft Corp; Redmond, WA) and checked for missing or implausible values. The primary outcome variables were lack of proficiency on PIKQ-UI and PIKQ-POP (scores of < 80 and < 50%, respectively). Continuous data were presented as means ± SD or median (IQR), and inter-clinic differences were assessed (Table 1) using ANOVA or Kruskal-Wallis, as appropriate. Categorical data were presented as percentages and compared using Chi-square testing; no cells were small enough to warrant Fisher’s exact test. Unadjusted ORs with 95% CIs were calculated on bivariate analysis comparing various covariates with knowledge non-proficiency on PIKQ-UI and PIKQ-POP scales. Adjusted ORs with 95% CI were calculated using multivariate logistic regression starting with the addition of all relevant and statistically significant covariates on bivariate analysis. Combined forward and backward stepwise regression was used to select the final model, with *p*-value ≤ 0.05 defining the criteria for inclusion. Covariates commonly thought of or previously shown to be confounders (i.e. age, race, income, and education) were locked into the model. List-wise deletion was performed for missing data in the logistic regression so that the entire record was excluded from analysis if relevant values were missing. Variance inflation factor (VIF) was performed on the covariates in the model to check for collinearity. Any variables with VIF ≥ 5 were removed from the final model. As there were many combinations of covariates relative to the sample size, logistic regression diagnostics were performed using the Hosmer and Lemeshow’s goodness-of-fit test. Data analyses was performed using Stata Statistical Software: Release 13 (Stata Corp; College Station, TX 2013).

**Table 1:** Baseline characteristics by clinic location

Variable	Geriatric (*n* = 113)	Community-based Primary Care (*n* = 128)	Hospital-based Primary Care (*n* = 105)	Total (*n* = 346)	*p*-value*
PIKQ Scores					
PIKQ-UI					
Median % (IQR)	58 (33–75)	58 (17–83)	58 (25–83)	58 (25–83)	0.87
Mean % ± SD	54 ± 31	52 ± 35	51 ± 35	53 ± 34	
PIKQ-POP					
Median % (IQR)	42 (8–75)	33 (8–67)	42 (8–75)	42 (8–67)	0.64
Mean % ± SD	43 ± 32	40 ± 33	42 ± 33	42 ± 33	
Knowledge					
PIKQ-UI, n (%)					
Non-proficient	86 (76.1)	88 (68.1)	75 (71.4)	249 (72.0)	0.52
PIKQ-POP, n (%)					
Non-proficient	58 (51.8)	70 (54.7)	57 (54.3)	185 (53.6)	0.99
Demographics					
Age, Mean ± SD	76 ± 10	51 ± 14	52 ± 15	60 ± 18	**< 0.001**
Age (by decade), n (%)					
18–29	0 (0.0)	9 (7.0)	12 (11.8)	21 (6.1)	
30–39	0 (0.0)	18 (14.1)	10 (9.8)	28 (8.2)	
40–49	2 (1.8)	28 (21.9)	20 (19.6)	50 (14.6)	
50–59	8 (7.1)	34 (26.6)	24 (23.5)	66 (19.3)	**< 0.001**
60–69	14 (12.5)	27 (21.1)	23 (22.6)	64 (18.7)	
70–79	45 (40.2)	9 (7.0)	11 (10.8)	65 (19.0)	
≥ 80	43 (38.4)	3 (2.3)	2 (2.0)	48 (14.0)	
Education, n (%)					
≤ High school^#^	49 (43.8)	35 (27.6)	32 (31.1)	116 (33.9)	
Some college^#^	19 (17.0)	43 (33.9)	31 (30.0)	93 (27.2)	**0.02**
≥ College^#^	44 (39.3)	49 (38.6)	40 (38.8)	133 (38.9)	
Income, n (%)					
< $30,000	40 (44.4)	35 (29.4)	38 (40.9)	113 (37.4)	
$30,000 – 50,000	21 (23.3)	33 (27.7)	23 (24.7)	77 (25.5)	0.22
> $50,000	29 (32.2)	51 (42.9)	32 (34.4)	112 (37.1)	
Comorbidities					
# of comorbidities, Median (IQR)	1 (0–2)	1 (0–2)	1 (0–2)	1 (0–2)	0.64
Cardiac disease, n (%)					
Yes	24 (22.2)	11 (8.7)	11 (10.9)	46 (13.7)	**0.008**
Hysterectomy, n (%)					
Yes	50 (46.7)	40 (31.7)	38 (36.9)	128 (38.1)	0.06
HRT, n (%)					
Yes	42 (40.8)	34 (34)	16 (18.0)	92 (31.5)	**0.003**
Smoking, n (%)					
Yes	7 (7.1)	17 (15.7)	14 (14.6)	38 (12.5)	0.13
Caffeine, n (%)					
Yes	56 (50.9)	60 (48.4)	45 (44.1)	161 (47.9)	0.61
Sources of information:					
Provider, n (%)					
Yes	109 (99.0)	123 (98.4)	99 (98.0)	331 (98.5)	0.81
Pharmacy, n (%)					
Yes	40 (36.4)	42 (33.6)	41 (40.6)	123 (36.6)	0.55
Internet, n (%)					
Yes	37 (33.6)	68 (54.4)	59 (58.4)	164 (48.8)	**< 0.001**
Television, n (%)					
Yes	29 (26.4)	30 (24.0)	21 (20.8)	80 (23.8)	0.64
Awareness of:					
Urinary incontinence, n (%)					
Yes	80 (79.2)	91 (73.4)	66 (68.0)	237 (73.6)	0.20
Pelvic organ prolapse, n (%)					
Yes	54 (53.5)	53 (43.1)	44 (44.0)	151 (46.6)	0.25
Fecal incontinence, n (%)					
Yes	73 (72.3)	72 (58.5)	52 (51.5)	197 (60.6)	**0.009**
Diagnosis of:					
Urinary incontinence, n (%)					
Yes	62 (54.9)	58 (45.3)	38 (36.2)	158 (45.7)	**0.02**
Pelvic organ prolapse, n (%)					
Yes	30 (26.5)	15 (11.7)	10 (9.5)	55 (15.9)	**0.001**
Fecal incontinence, n (%)					
Yes	19 (17.0)	11 (8.6)	6 (5.7)	36 (10.4)	**0.02**
Any pelvic floor disorder, n (%)					
Yes	52 (46.0)	41 (32.0)	29 (27.6)	122 (35.3)	**0.01**

*Abbreviations*: *PIKQ* prolapse and incontinence knowledge questionnaire, *PFD* pelvic floor disorders, *UI* urinary incontinence, *POP* prolapse, *IQR* interquartile range, *HRT* hormone replacement therapy

*p-values are based on comparisons across clinic locations (e.g. geriatric vs. community-based primary clinic vs. hospital-based primary care clinic). Continuous data was compared using ANOVA or Kruskal-Wallis test, as appropriate based on whether data was parametric or non-parametric. Categorical data was compared using Chi-square test

^#^ ≥ College: at least college degree; ≤ High school: high school degree or less

Missing data for covariates: age (1.2%), parity (2.9%), race (6.9%), education (1.2%), income (12.7%), cardiac disease (2.9%), hysterectomy (2.9%), HRT (15.6%), smoking (12.4%), caffeine (2.9%), provider (2.9%), pharmacy (2.9%), internet (2.9%), television (2.9%), UI awareness (6.9%), POP awareness (6.4%), FI awareness (6.1%), UI diagnosis (1.5%), POP diagnosis (1.5%), FI diagnosis (1.5%), PFD diagnosis (1.5%). Questionnaires with incomplete PIKQ-UI or –POP questionnaires were excluded

Bolded *p*-values are significant

## Results

Three hundred and forty-six women participated in this study: 32.7% from the geriatric clinic, 37.0% from the primary care community-based clinic, and 30.3% from the primary care hospital-based clinic (Table 1). Response rate could not be calculated as use of an openly distributed, self-administered questionnaire impaired our ability to determine the number of non-participants. Knowledge was similar across all three locations for both UI and POP. Only 28.0% of participants were UI proficient (defined as PIKQ-UI score ≥ 80%), and the median score was 58.0%. POP proficiency was higher at 46.4% (defined as PIKQ-POP score ≥ 50%), with a median score of 42.0%. Respondent’s mean age was 60 (range: 21–97), with the geriatric-clinic cohort significantly older (mean: 76 years) than the community-based (mean: 51 years) and hospital-based contingents (mean: 52 years). Geriatric-clinic participants were also significantly more parous, less educated, used the internet less as a source of medical information, had more cardiac comorbidities, had higher usage of hormone-replacement therapy, were more aware of FI as a medical condition, and had higher rates of PFD diagnosis. The vast majority of respondents were either white (64.3%) or African-American (28.3%), with only 7.5% classifying themselves as “Other”, which included Asians and Hispanics. Significantly more African-American participants came from the hospital-based clinic (44.0%) than from the community-based (25.6%) or geriatric clinics (15.2%). All other demographic and clinical characteristics were consistent across clinic cohorts.

To assess for associations with PIKQ-UI and -POP proficiency, bivariate analyses were conducted on demographic and clinical factors, as well as awareness of PFDs as medical conditions (Table 2). Women of certain age groups (40–49, ≥80 years), African-American descent, lower educational attainment, income of <$30,000/year, or women who did not use the internet as a health-information source were significantly more likely to be UI and POP knowledge non-proficient. POP knowledge non-proficiency was also associated with not drinking caffeine. Unsurprisingly, prior PFD diagnoses held significant associations: no previous UI diagnosis was significant for UI knowledge non-proficiency, and no diagnosis of either POP or any PFDs were associated with POP knowledge non-proficiency. Being unaware of any PFDs as a medical condition—be it UI, FI, or POP—was also significantly associated with knowledge non-proficiency for both UI and POP.

**Table 2:** Bivariate analysis of variables associated with UI and POP knowledge

	Lack of knowledge on PIKQ-UI scale (< 80% correct)			Lack of knowledge on PIKQ-POP scale (< 50% correct)		
	OR	95% CI	*p*-value	OR	95% CI	*p*-value
Age, years						
18–29	1.84	0.63–5.34	0.26	1.24	0.46–3.32	0.67
30–39	2.21	0.83–5.92	0.11	2.38	0.94–6.03	0.07
40–49	2.33	1.04–5.26	**0.04**	2.19	1.03–4.68	**0.04**
50–59	1.00	ref		1.00	ref	
60–69	1.88	0.91–3.91	0.91	0.94	0.47–1.86	0.85
70–79	1.92	0.93–3.99	0.93	0.75	0.38–1.50	0.42
≥ 80	3.68	1.49–9.08	**0.005**	2.66	1.21–5.87	**0.01**
Education						
≥ College	1.00	ref		1.00	ref	
Some college	1.41	0.82–2.45	0.22	1.81	1.06–3.10	**0.03**
≤ High school	6.73	3.31–13.70	**< 0.001**	5.50	3.16–9.57	**< 0.001**
Income						
> $50,000	1.00	ref		1.00	ref	
$30,000 - 50,000	1.30	0.70–2.39	0.41	1.19	0.66–2.13	0.56
< $30,000	3.08	1.65–5.75	**< 0.001**	2.41	1.40–4.13	**0.001**
Race						
White	1.00	ref		1.00	ref	
African-American	2.87	1.49–5.53	**0.002**	2.29	1.37–3.86	**0.002**
Other	0.48	0.20–1.12	0.09	0.65	0.27–1.56	0.34
Caffeine						
Yes	1.00	ref		1.00	ref	
No	1.42	0.88–2.30	0.15	1.79	1.16–2.76	**0.009**
Information						
Yes: Internet	1.00	ref		1.00	ref	
No: Internet	2.38	1.46–3.87	**< 0.001**	2.30	1.49–3.57	**< 0.001**
Awareness of PFDs						
Yes	1.00	ref		1.00	ref	
No: UI	6.70	2.96–15.17	**< 0.001**	6.76	3.65–12.50	**< 0.001**
No: POP	4.13	2.46–6.92	**< 0.001**	13.05	7.68–22.17	**< 0.001**
No: Fecal incontinence	5.93	3.18–11.05	**< 0.001**	5.62	3.41–9.25	**< 0.001**
Diagnosis of PFDs						
Yes	1.00	ref		1.00	ref	
No: UI	1.96	1.09–3.55	**0.03**	1.54	0.86–2.73	0.14
No: POP	1.71	0.92–3.21	0.25	2.10	1.13–3.89	**0.02**
No: FI	1.51	0.75–3.05	0.09	1.40	0.72–2.74	0.32
No: UI, FI, and POP	1.43	0.88–2.32	0.15	1.66	1.06–2.59	**0.03**

As age as a continuous variable did not have a linear relationship with the log odds of our outcomes, it was categorized according to decades of age

*Abbreviations*: *PIKQ* prolapse and incontinence knowledge questionnaire, *PFDs* pelvic floor disorders, *UI* urinary incontinence, *POP* prolapse, *FI* fecal incontinence, *ref*. referent

Bolded *p*-values are significant

The only factors to remain independently associated with UI knowledge non-proficiency on multivariate analysis were age group 40–49 years, low levels of education (educational attainment of high school diploma or less), being unaware that UI or FI are medical conditions, and having no prior diagnosis of UI (Table 3). The final model excluded being unaware of POP and no prior diagnosis of POP or FI due to collinearity (VIF ≥ 5) with being unaware of UI and FI, and no previous diagnosis of UI. The only factors to achieve independent associations with POP knowledge non-proficiency on multivariate analysis were age group 30–39 years and being unaware of UI or POP as medical conditions. The final model excluded being unaware of FI and no previous diagnosis of UI, POP, and FI due to collinearity (VIF ≥ 5). It is interesting to note that both no previous diagnosis of UI and being unaware of UI remained in the final UI knowledge non-proficiency model, but only lack of POP awareness was included in the POP knowledge non-proficiency model; participants who were aware of POP were more likely to be diagnosed with POP whereas individuals who were aware of UI may not have had a previous diagnosis of UI. Several logistic regression diagnostics were performed as detailed in Methods and no problems were identified.

**Table 3:** Multivariate analysis of variables associated with UI and POP knowledge

	Lack of knowledge on PIKQ-UI scale (< 80% correct)			Lack of knowledge on PIKQ-POP scale (< 50% correct)		
	OR	95% CI	*p*-value	OR	95% CI	*p*-value
Age, years						
18–29	1.85	0.44–7.75	0.40	0.52	0.10–2.60	0.43
30–39	2.43	0.73–8.19	0.15	3.89	1.03–14.64	**0.04**
40–49	3.32	1.06–10.39	**0.04**	3.00	0.94–9.65	0.07
50–59	1.00	ref		1.00	ref	
60–69	2.25	0.80–6.30	0.12	1.24	0.39–3.96	0.72
70–79	2.56	0.92–7.08	0.07	1.12	0.38–3.29	0.84
≥ 80	2.08	0.59–7.36	0.26	2.94	0.77–11.20	0.12
Education						
≥ College	1.00	ref		1.00	ref	
Some college	0.96	0.45–2.09	0.93	1.27	0.55–2.96	0.58
≤ High school	3.24	1.10–9.61	**0.03**	1.64	0.61–4.40	0.32
Income						
> $50,000	1.00	ref		1.00	ref	
$30,000 - 50,000	0.79	0.34–1.82	0.59	0.87	0.35–2.14	0.76
< $30,000	1.85	0.78–4.42	0.17	1.06	0.43–2.64	0.90
Race						
White	1.00	ref		1.00	ref	
African-American	2.02	0.94–4.37	0.22	2.02	0.99–4.09	0.05
Other	0.36	0.12–1.11	0.12	0.81	0.23–2.77	0.95
Internet for information						
No	1.00	ref		1.00	ref	
Yes	1.14	0.56–2.32	0.72	1.23	0.57–2.64	0.60
Awareness of PFDs						
Yes	1.00	ref		1.00	ref	
No: UI	3.58	1.10–11.70	**0.043**	3.94	1.68–9.23	**0.002**
No: POP	removed from the model*			10.05	5.26–19.18	**< 0.001**
No: Fecal incontinence	3.29	1.34–8.07	**0.01**	removed from the model**		
Diagnosis of PFDs						
Yes	1.00	Ref		1.00	ref	
No: UI	2.48	1.13–5.45	**0.045**	–		
No: POP		–		removed from the model**		
No: FI		–		–		
No: UI, FI, and POP		–		removed from the model**		

Multivariate models were used to estimate the adjusted OR for the lack of proficiency in urinary incontinence and prolapse knowledge using a combination of forward and backward stepwise regression locking in age, race, income, and education. As age as a continuous variable did not have a linear relationship with the log odds of our outcomes, it was categorized it into decades of age

The urinary incontinence knowledge multivariate analysis included 209 subjects (74% of data) and the prolapse knowledge analysis included 215 subjects (75% of data) who had values for all the covariates

*Abbreviations*: *PIKQ* prolapse and incontinence knowledge questionnaire, *PFDs* pelvic floor disorders, *UI* urinary incontinence, *POP* prolapse, *FI* fecal incontinence, *ref*. referent, *VIF* variance inflation factor

“—” Variables that did not achieve significance on bivariate analysis and so were not included for consideration in the multivariate model

*Being unaware of POP and no prior diagnosis of POP and FI were excluded from the final model due to collinearity (VIF ≥ 5)

**Being unaware of FI and no prior diagnosis of UI, POP, FI were excluded from the final model due to collinearity (VIF ≥ 5)

Bolded *p*-values are significant

## Discussion

In this population of women seeking primary care, there is a considerable dearth of PFD knowledge. Based on proficiency criteria established by the PIKQ authors, 72% of our respondents were UI knowledge non-proficient and 54% were POP knowledge non-proficient. These estimates are similar to those from two earlier studies—one in a gynecology setting and one in a community-based setting—which used the same questionnaire and cut-off scores (UI knowledge non-proficiency: 62–81%, POP knowledge non-proficiency: 48–76%) [19, 20]. This finding suggests that patient PFD knowledge is similarly low regardless of when or where patients are recruited, although all studies were conducted in urban settings near major academic centers.

Improving PFD knowledge is critical as such knowledge has been linked to patients seeking care earlier in the disease process [11, 24–28]. In turn, such early presentation to care allows for more conservative, non-surgical treatment options, such as Kegel exercises and pessary placement, which have been proven effective and may even delay disease progression [22, 23]. This rationale is why our study targeted women presenting for primary care. Although this population is connected to the healthcare system, they may not readily volunteer issues related to PFDs with their healthcare professionals due to embarrassment, belief that they are simply “a normal part of aging,” or being unaware that treatment options exist [9, 11–14]. Importantly, increasing knowledge of these conditions has also been demonstrated to increase compliance with treatments [29].

On bivariate analysis, several demographic factors (i.e. 40–49 and ≥ 80 years age groups, African-American race, income <$30,000/yr., lower educational attainment) were significantly associated with both UI and POP knowledge non-proficiency. However, only 40–49 years age group and educational attainment in the UI model retained significance on multivariate analysis. The literature contains mixed results regarding associations between race and PFD knowledge. Our findings are consistent with a number of studies that found no association between race and PFD knowledge, but it is worth noting that other studies report decreased knowledge in African-Americans after accounting for socioeconomic and educational factors [18–21, 29]. One study reporting such racial differences in knowledge was specifically designed to evaluate for race-based differences and consequently, consisted of over 50% African-American participants. Although 28% of our population was African-American, we may have lacked sufficient power to detect more subtle differences in PFD knowledge. It is also worth noting the low representation of Hispanic women in our study compared to national levels. The patient populations in the clinics surveyed are predominantly Caucasian and African-American and our questionnaire was provided solely in English. There is also evidence that Hispanics are significantly more likely to lack a primary healthcare provider than non-Hispanic blacks or whites [30].

Education appears to be a discrete factor in PFD knowledge. While those with lower educational attainment were more likely to lack UI and POP knowledge, on multivariate analysis, this variable only remained significant for UI knowledge non-proficiency. In a 2015 survey of community-dwelling women, Mandimika et al. used the same questionnaire and cut-off scores, and found that low educational attainment was also independently associated with UI knowledge non-proficiency, but not POP knowledge non-proficiency [29]. The reason for this difference remains unclear. In our population, the strongest predictor of POP non-proficiency was a self-reported lack of awareness of POP as a medical condition. However, the same was true for awareness of UI in predicting UI knowledge non-proficiency, although the magnitude was similar to that of lower educational attainment (OR: 3.58 vs. 3.24). This may suggest that while education may increase awareness of PFDs as medical conditions, education and awareness remain separate entities that may contribute uniquely to UI and POP knowledge.

Awareness of PFDs as medical conditions ranged from 46.6% for POP to 73.6% for UI, with FI in between at 60.6%. A study assessing women’s understanding of urogynecologic terminology found similar results, with 80% UI awareness and 52% POP awareness [31]. These differences in awareness between PFDs may result from the use of phrases like “urinary incontinence” and “fecal incontinence” in common vernacular, whereas “pelvic organ prolapse” is generally less used [31]. The use in common vernacular may also mean that while “urinary and fecal incontinence” are more commonly heard, there may be more misconceptions surrounding these terms; whereas women who reported POP awareness truly possessed POP knowledge. This is also consistent with our finding that 37.6% of respondents who reported UI awareness actually demonstrated knowledge proficiency on PIKQ-UI, while 78.2% who reported POP awareness were knowledge proficient on PIKQ-POP. Therefore, while being unaware of a PFD as a medical condition was strongly associated with knowledge non-proficiency, this relationship was stronger for POP than for UI as UI unaware respondents were 3.6 times more likely to be UI knowledge non-proficient and POP unaware respondents were 10.1 times more likely to be POP knowledge non-proficient.

These findings may serve to inform various teaching strategies for PCPs. Given the strong correlation between unawareness of PFDs as medical conditions and UI/POP knowledge non-proficiency, one simple screening strategy could be to ask patients if they are aware that UI, FI, and POP are medical conditions with treatment options and not simply “a normal part of aging or childbearing.” If the patient is unaware, then PCPs may provide a brief explanation of these conditions, provide educational materials, and emphasize that these are medical conditions with effective non-surgical and surgical treatment options. Such proactive measures by PCPs may lead patients to open up about these symptoms when embarrassment or other barriers may have otherwise prevented such conversations which may result in patients being referred to specialists at earlier stages of disease progression.

One limitation to this study is selection bias, in that participants were approached in primary care clinics and thus were not a truly community-based sample. These women were already active participants in their healthcare and likely had at least enough baseline medical knowledge to seek care. Additionally, participants completed the study questionnaire after either being approached by a study team member or on their own after seeing the questionnaire at advertised locations in the clinic. With either recruitment method, study participants’ decision to start and complete the questionnaire was done independently. On the cover sheet of our questionnaire, it was clearly stated that this is a survey on knowledge of PFDs including UI and POP; therefore, the women that chose to complete the questionnaire may be women that were already aware of or have been previously diagnosed with these conditions. However, our proficiency estimates were similar to other studies with different study designs and study populations, including community-based studies [18–21]. Additionally, the prevalence of PFDs in our population is comparable to other studies (10–46%), making selection bias according to disease prevalence less likely. Our study’s racial composition was not comparable to national averages. We were limited by the number of Hispanic and Asian women, which are populations that are potentially more affected by PFDs than African-American and Caucasian women [29, 32–34]. Despite the possibility of increased PFD prevalence, other studies in these specific populations have also reported a lack of knowledge regarding PFDs [21, 35–37].

## Conclusions

There is a lack of knowledge of PFDs in women presenting for primary care, and different approaches should be taken to increase patients’ UI and POP proficiency. For UI, healthcare providers should assess patients’ actual understanding of the disease, especially among those with lower educational attainment, to eliminate any possible misconceptions. For POP, the focus should be on increasing awareness of this disease, as many women may have not previously heard of this condition. Increased awareness and knowledge of PFDs can lead to increased care seeking for these conditions [11, 24–28]. While imparting knowledge can be time-consuming, simple strategies to communicate that PFDs are not a “natural part of childbirth and aging” can alter perception and knowledge. This is especially pertinent in primary care settings as these are often patients’ “first line of defense” in facilitating their access to other health care services and specialists. Furthermore, addressing conditions such as UI and POP at earlier stages of disease progression may also allow for more treatment options, especially conservative options such as Kegel exercises and pessary placement [22, 23]. Early care also decreases the risk of developing potentially consequential sequelae such as urinary retention, which can lead to urinary tract infections, stone development, and kidney damage. As such, it is essential that the timeliness of care-seeking for PFDs be improved among women, which may be facilitated by increasing awareness and knowledge of these conditions.

## Abbreviations

FI: Fecal incontinence
IRB: Institutional Review Board
PCP: Primary care provider
PDF: Pelvic floor disorder
PIKQ: Prolapse and Incontinence Knowledge Questionnaire
POP: Pelvic organ prolapse
UI: Urinary incontinence
VIF: Variance Inflation Factor
